# A Novel *COCH* p.D544Vfs*3 Variant Associated with DFNA9 Sensorineural Hearing Loss Causes Pathological Multimeric Cochlin Formation

**DOI:** 10.3390/life14010033

**Published:** 2023-12-25

**Authors:** Yingqiu Peng, Mengya Xiang, Ting Fan, Xiaofang Zhong, Aqiang Dai, Jialing Feng, Pengfei Guan, Jiamin Gong, Jian Li, Yunfeng Wang

**Affiliations:** 1 ENT Institute and Department of Otorhinolaryngology, EYE & ENT Hospital, Fudan University, Shanghai 200031, China; 2 NHC Key Laboratory of Hearing Medicine, Fudan University, Shanghai 200031, China; 3 Clinical Laboratory Center, Children’s Hospital of Fudan University, Shanghai 201102, China

**Keywords:** DFNA9, hearing loss, genetics of hearing loss, cochlin

## Abstract

*COCH* (coagulation factor C homology) is one of the most frequently mutated genes of autosomal dominant non-syndromic hearing loss. Variants in *COCH* could cause DFNA9, which is characterized by late-onset hearing loss with variable degrees of vestibular dysfunction. In this study, we report a Chinese family with a novel *COCH* variant (c.1687delA) causing p.D544Vfs*3 in the cochlin. Comprehensive audiometric tests and vestibular function assessments were taken to acquire the phenotypic profile of the subjects. Next-generation sequencing was conducted and segregation analysis was carried out using Sanger sequencing. The proband presented mild vestibular symptoms and normal functional assessment results in almost every test, while the variant co-segregated with hearing impairment in the pedigree. The variant was located beyond the vWFA2 domain, which was predicted to affect the post-translational cleavage of the cochlin via molecular modeling analysis. Notably, in the overexpressing study, by transient transfecting the HEK 293T cells, we found that the p.D544Vfs*3 variant increased the formation of multimeric cochlin. Our result enriched the spectrum of DFNA9-linked pathological *COCH* variants and suggested that variants, causative of cochlin multimerization, could be related to DFNA9 with sensorineural hearing loss rather than serious vestibular symptoms.

## 1. Introduction

The prevalence of hereditary hearing loss is approximately 1/1000 in live births, among which 70% of the patients have non-syndromic hearing loss [1]. The etiology of hereditary hearing loss includes both genetic and environmental factors [2]. To date, more than 120 non-syndromic hearing loss genes have been identified, approximately 40% of which are autosomal dominant non-syndromic hearing loss (ADNSHL) genes [3]. Although hereditary hearing loss is known to be highly clinical/genetically heterogeneous, a majority of ADNSHL is post-lingual and progressive hearing loss, such as DFNA9, DFNA22, and DFNA36 [4]. Patients with these ADNSHL types can be identified during early stages and treated to prevent hearing loss onset or progression, which sheds light on the importance of molecular biological diagnosis of hearing loss.

*COCH* is one of the most frequently mutated genes of ADNSHL [5]. The pathogenic variants in the *COCH* gene could cause DFNA9, which is typically late-onset and associated with variable vestibular dysfunction evolving bilateral vestibulopathy, causing, among other things, gait imbalance, oscillopsia, spatial disorientation, and increased risk of falling [6,7]. Although the exact prevalence of DFNA9 is unknown, it has been reported that DFNA9 was found in several families on four continents. According to studies on *COCH*, the prevalence of *COCH* variants in ADNSHL patients in the European population is higher than in Asia, but this may be due to the higher detection rate in the European population [6,8]. *COCH* gene encodes a secreted protein named cochlin, the major noncollagenous protein in the extracellular matrix of cochlea and vestibular labyrinth [8,9]. Cochlin contains an N-terminal signal peptide (SP), an LCCL (Limulus factor C, cochlin, lung gestational protein) domain, and two vWFA (von Willebrand factor A-like) domains [10]. The LCCL domain consists of a central α-helix enclosed by two β-sheets, which is an autonomously folding domain thought to assist host defense functions [11,12,13]. The vWFA domains are integral components of many secreted and extracellular matrix proteins, and they bind other proteins, such as glycoproteins, proteoglycans, and fibrillar collagens [14,15,16]. DFNA9 hearing loss occurs with different pathogenesis and clinical features depending on variant location. Most DFNA9-related variants were located in the LCCL or vWFA-like domains [5]. It has been proven that missenses and deletion variants in the LCCL domain can result in misfolding of this domain [17], which may hide the binding site for aggrecanase-1, making it less accessible for cleavage [18]. Furthermore, coupling of variant cochlins and heterodimer formation of wild-type and variant cochlins is induced by some LCCL domain variants [19]. Currently, several studies have demonstrated that vWFA domain variants also cause high-molecular-weight cochlin aggregation in cells [20]. However, recent studies detected *COCH* variants at the protein’s C-terminus that co-segregated with DFNA9 hearing impairment in pedigrees, suggesting that proper function of the non-domain region of cochlin was also required for normal hearing [21,22,23]. These studies also reported that ADNSHL patients with *COCH* p.I541F, p.C542Y, and p.C542F variants have no vestibular symptoms [24]. Nevertheless, little is known about the correlation between Chinese DFNA9 families and C-terminus variants of cochlin protein. In this study, we identified a novel variant at the C-terminus of cochlin protein in a Chinese DFNA9 family and expanded on the above findings.

## 2. Materials and Methods

### 2.1. Subjects

One Chinese family (Family621) experiencing ARNSHL was ascertained for this study. Among the 29 individuals in Family621, eight (II.1, II.2, II.3, II.5, II.7, III.3, III.4, and IV.3) underwent clinical examination, pure tone audiometry, and genetic testing in a specialized hospital. The proband, i.e., III.3, also took vestibular function tests due to her vertigo complaint. The hearing condition of the other family members was assessed by inquiring about disease history through a telephone conversation with the individuals themselves or with their relatives. Hearing thresholds (at 125, 250, 500, 1k, 2k, 4k, and 8k Hz) were measured in a calibrated soundproof room (self-noise < 30 dB SPL A). The pure tone average (PTA) at 500, 1k, 2k, and 4k in the better ear was calculated and used for hearing loss classification [25]. This study was approved by the Ethics Committee of Eye and ENT Hospital of Fudan University (approval number: 2017044).

### 2.2. Vestibular Function Assessment

To evaluate the vestibular function of participants who complained of a history of vestibular symptoms, the bithermal caloric test, rotary chair test, video head-impulse test (vHIT), cervical vestibular evoked myogenic potential (VEMP) test, and sensory organization test (SOT) were applied.

In the bithermal caloric test, the subject lay on their back with their head leaned forward by 30°. The subject’s bilateral external canals were alternately irrigated with 50 mL of cold water (30 °C) and hot water (44 °C) constantly for 40 s each time. During this period, binocular nystagmus was recorded via videonystagmography (VNG). The irrigations were performed following a regular stimulation sequence, which is usually right cold (RC), left cold (LC), right warm (RW), and left warm (LW), with an interval of at least 5 min between each irrigation. Unilateral weakness (UW) and directional preponderance (DP) were calculated using the following equation:

DP = [(RW + LC) − (LW + RC)]/(RW + RC + LW + LC) × 100%

UW = [(RW + RC) − (LW + LC)]/(RW + RC + LW + LC) × 100%

RW, RC, LW, and LC represent the maximum nystagmus slow-phase angular velocity induced by hot air in the right ear, cold air in the right ear, hot air in the left ear, and cold air in the left ear, respectively.

The participant was assessed via the rotatory chair test through a rotatory chair system. The test was performed in a dark room. Participants were asked to sit on a rotating chair with their heads tilted 30° forward and restrained in order to make sure the horizontal semicircular canals were close to the plane of rotation. The test includes both clockwise and counterclockwise rotations. VNG was used to record the evoked eye movements.

In the vHIT, corrective saccades were recorded when the subject rapidly turned their head to the side of vestibular weakness. Each side received about 20 manual horizontal head impulses with random timing and direction. Video-oculography was concurrently captured on the same eye. To demonstrate the accuracy of the computed gains, two datasets were gathered for each recording session. Online video image analysis utilizing a pupil detection technique was used to determine eye position and velocity. A normal vestibular ocular reflex (VOR) velocity gain was defined as being 0.7 or higher for the anterior and posterior semicircular canal, or 0.8~1.2 for the horizontal semicircular canal according to practitioner protocol.

A 500 Hz sound was used as a stimulus and was presented to the subject monaurally through air conduction by inserting earphones. Surface electrodes were placed at the midpoint of the ipsilateral Sternocleidomastoid Muscle (SCM) to record the evoked C-VEMPs, while the electrode on the upper part of the sternum was the reference electrode, and the one on the forehead was used as a ground electrode. The subjects were instructed to lift their heads while tightening their chins. Biphasic waves accepted in 30 msec after the stimulus which initiated with positive polarity (P1) and then followed with negative polarity (N1) were considered positive C-VEMP responses.

In SOT, the EquiTest Balance Master Systems (NeuroCom, Clackamas, OR, USA) was operated as described in previous research [26]. During the examination, the subjects stood on the platform under effective protection, and were required to maintain their body balance as much as possible. Tests of six conditions (C1–C6) were carried out from easy to difficult, and each condition was performed 3 times for 20 s each time, and the mean value was recorded. The system software scored the equilibrium score (ES) [26] for each test:

ES = {1 − [θmax(A) − θmax(P)]/12.5} × 100

θmax(A) is the maximum forward sway angle in the 20 s duration, θmax(P) is the maximum backward sway angle, and 12.5° is the theoretical maximum of sway in the sagittal plane for normal stance of an individual [27]. ES is the percentage of the actual maximum swing amplitude to the theoretical swing limit, with a score close to 100 indicating that the swing is very small, and a score of 0 indicating that the swing is close to the upper limit. Among the six SOT conditions, C1-C3 was tested under the conditions of open eyes, closed eyes, and open eyes + visual shaking when the support platform was stable and motionless, and C4-C6 was tested under the conditions of open eyes, closed eyes, and open eyes + visual shaking when the support platform was active. According to the changes in the body’s center of gravity under the six conditions, the SOT not only routinely reflects the comprehensive balance ability of the human body, but also evaluates the independent weights of vestibulo, visual, and proprioceptive sensory functions in the adjustment of body balance. The SOT results consisted of four parts: ES and composite ES histograms, sensory weight histograms, sensory strategies, and center of gravity (COG) analysis for the six tests. The ES is between 0~100; the better the balance function, the higher the score. If the subject takes a step during the test, they are considered to have fallen and are awarded 0 points. Composite ES refers to the weighted average score of the six ESs under C1-C6, which reflects the comprehensive ability of the balance center to use vestibular, proprioceptive, and visual information to carry out sensory integration and maintain body balance, and its score ≥ 70 is in the normal range and abnormal at <70. SOT sensory weights reflect the changes in sensory weights of vestibular, proprioceptive, and visual sensations in the process of balancing sensory integration. In this study, the SOT results were judged to be abnormal if there were any abnormalities in the composite ES < 70 and/or the sensory weights of the vestibule, proprioception, and vision.

### 2.3. Next-Generation Sequencing and Bioinformatic Analysis

Peripheral blood samples were collected from subjects of the pedigree (II 1, II 2, III 3, III 4, and IV3) and a healthy volunteer after obtaining informed consent. Genomic DNA was extracted using the TIANamp Blood DNA Kit (TIANGEN, Beijing, China) based on the protocols recommended by the manufacturer. The DNA samples of all subjects were used in whole-exome sequencing. The whole-exome library preparation and next-generation sequencing were conducted using the Illumina HiSeq 2000 platform. After that, 100 bp paired-end sequencing was performed, providing each sample had at least 80-fold coverage. Illumina’s Cassava pipeline with default settings was used to process raw image files for base-calling. After filtering out low-quality reads (unknown bases > 5), the reads were aligned to the NCBI human reference genome (hg19) using the Burrows-Wheeler Aligner (BWA) program, followed by local realignment and base quality assessment using Genome Analysis Toolkit (GATK) according to the Broad best practices pipeline [28,29]. ANNOVAR [30] software was used for variant identification and annotation. To identify pathogenic variants in the study, annotated variants went through several filtering steps, as the previous study described [31]. First, we excluded low-quality variants with a Depth <10 or Genotype Quality < 30. Second, variants in noncoding regions were dropped. Third, synonymous variants in the coding region were removed. Fourth, we filtered out variants with a minor allele frequency (MAF) > 0.001 in several databases (1000 Genome Project, GnomAD v2.1.1, and an in-house database). The deleterious effect of variants was predicted based on the CADD score. To validate the variants, segregation analysis was carried out using Sanger sequencing according to previous studies [32,33]. A single primer 5′-TCTTCTCTGTTGGTGTGGCTTG-3′ was used in Sanger sequencing, performed on a 3730XL sequencer (Applied Biosystems, Foster City, CA, USA) according to the manufacturer’s instructions. Nucleotide sequence data obtained via Sanger sequencing will be available in the GenBank databases under the accession numbers OR687462, OR687463, OR687464, OR687465, and OR687466. Multiple sequence alignments were performed using T-coffee (https://tcoffee.crg.eu, accessed on 5 December 2021) with the default setting of six species. Firstly, sequences of *COCH* of homo sapiens, bos taurus, mus musculus, rattus norvegicus, danio rerio, and gallus were downloaded from Uniprot (https://www.uniprot.org/, accessed on 5 December 2021), and the proper format of the sequence was adjusted for T-coffee (https://tcoffee.crg.eu, accessed on 5 December 2020). After the above sequences were input to T-coffee, a conservation analysis of six species was obtained. The variant site was identified using the Ensemble database (https://asia.ensembl.org/index.html, accessed on 5 December 2021).

### 2.4. Molecular Modeling and Binding Ligand Prediction

*COCH* wild type (GenBank: AAQ89259.1) and mutant protein amino acid sequences were used to obtain predicted protein models from the I-TASSER online server (https://zhanggroup.org/I-TASSER/, accessed on 5 December 2021) [34]. Among the five predicted proposed models, the model with the highest cluster density and confidence score, which were calculated by I-TASSER to estimate the quality of predicted models, was selected as the final protein model. BioLiP was used for screening possible binding ligands, followed by ligand binding site prediction using the COACH algorithm [35]. The PyMOL Molecular Graphics System (version 2.5.0; software available at http://www.pymol.org, accessed on 5 December 2021) was used for model visualization.

### 2.5. Cell Culture and Plasmid Transfection

For convenience of detection, the 3xHA fragment was inserted before the stop codon of *COCH* protein-coding sequence (NCBI human cDNA reference sequence: NM_004086.2). The wild-type and mutant *COCH*-3xHA sequences were synthesized by GENEWIZ (Suzhou, China), cloned into a vector, and driven by the CAG promoter. Colonies were selected and grown, and plasmid DNA was harvested using the Plasmid Plus Midi Kit (QIAGEN, Hilden, Germany). After sequence confirmation, wild-type and mutant *COCH* plasmids were used for cell transfection, respectively.

Dulbecco’s modified essential medium (DMEM) (SH30023.01, HyClone, Marlborough, MA, USA) together with 10% fetal bovine serum (abs972, Absin, Shanghai, China) and penicillin (50 IU/mL)/streptomycin (50 μg/mL) (V900929, Sigma-Aldrich, Burlington, MA, USA) was used to culture Human embryonic kidney (HEK) 293T cells. Cells were incubated in an incubator that maintained a temperature of 37 °C and 5% CO_2_ in humidified air. According to the manufacturer’s instructions, after 24 h’ culture, when the number of HEK293T cells was appropriate, HEK293 cells were transfected with wild-type or mutant *COCH* plasmids in Opti-MEM™ Reduced Serum Medium (31985070, Thermo Fisher Scientific, Waltham, MA, USA) using Lipofectamine 3000 (L3000-015, Invitrogen, Waltham, MA, USA). Protein was collected from HEK293T cells after 48 h’ culture, while Immunofluorescence was operated after 72 h’ incubation, during which HEK293T cells were cultured in DMEM with 10% fetal bovine serum but without penicillin/streptomycin.

### 2.6. Cell Viability Assay

Cell viability was gauged by the Cell Counting Kit-8 (CK04-500T, Dojindo, Mashiki, Japan), which was used according to the manufacturer’s instructions. HEK 293T cells were seeded in 96-well plates with 5 × 10^3^ cells per well after plasmid transfection and were incubated overnight in appropriate conditions in three replicates. CCK-8 was added to every well for 4 h in 100 µL culture medium. Optical density (OD) at 450 nm of each group was quantified using a microplate reader (Bio-Rad, Hercules, CA, USA). The positive control was the same process without cell seeding, while the negative control was without drug treatment. The relative viability was calculated according to the formula:

(OD_experiment_ − OD_positive_)/(OD_negative_ − OD_positive_) × 100.

### 2.7. Protein Extraction and Western Blot

Ice-cold RIPA lysis buffer (HY-K1001, MedChemExpress, Shanghai, China) with protease inhibitor cocktail (HY-K0010, MedChemExpress, Shanghai, China) was used to lyse cells for 30 min at 0 °C, and the mixture was shaken vigorously three times during the process. The lysed cells were centrifuged at 12,000× *g* for 20 min at 4 °C. The concentration of protein was checked using the BCA protein assay kit (P0010S, Beyotime Institute Biotechnology, Shanghai, China) after the supernatant was collected. Then, protein was boiled for 5 min at 95 °C with 5×SDS-PAGE protein loading buffer under reducing condition, whereas 5 × SDS-PAGE protein loading buffer (non-reducing) was used for the non-reducing condition. Equal amounts of protein samples were loaded and ran through 10% SDS-PAGE gel under reducing condition, or through 8% SDS-PAGE for non-reducing condition. The separated proteins were transferred to polyvinylidene difluoride membranes (IPVH00010, Millipore, Burlington, MA, USA) under a constant 250 mA current. Next, 5% non-fat dried milk in Tris-buffered saline involving 0.1% Tween-20 (TBST) (G0004-1L, Servicebio, Wuhan, China) was applied to block the membranes at room temperature for 1 h. And the membranes were incubated with the primary antibodies in TBST, which included 5% non-fat dried milk at 4 °C overnight. The primary antibodies were anti-HA (1:1000 dilution; C29F4, Cell Signaling Technology, Danvers, MA, USA) and anti-GAPDH (1:10,000 dilution; 60004-1-Ig, Proteintech, Rosemont, IL, USA). HRP-Goat Anti-Rabbit IgG (H+L) (1:2500 dilution; 111-035-003, Jackson ImmunoResearch, West Grove, PA, USA) and HRP-Goat Anti-Mouse IgG (H+L) (1:2500 dilution; 115-035-003, Jackson ImmunoResearch, USA) were used as the secondary antibodies separately.

### 2.8. Immunofluorescence

HEK 293T cells were fixed with 4% PFA for 5 min and then were washed out three times with PBS. After that, these samples were blocked with 0.5g Donkey Serum Albumin in PBST (1% Triton X-100 mixed with PBS) for 1 h followed by incubation with the anti-HA antibody (1:500 dilution; C29F4, Cell Signaling Technology, Danvers, MA, USA) overnight at 4 °C. The next day, the secondary fluorescent antibody Alexa Fluor^®^ 488-AffiniPure Donkey Anti-Rabbit IgG (H+L) (1:500 dilution; 711-545-152, Jackson ImmunoResearch, West Grove, PA, USA) was used to incubate these samples for 1 h at room temperature, avoiding light. Nuclei were labeled with Hoechst (DAPI; D9542, Sigma-Aldrich) for 3 min, avoiding light, at room temperature, after the samples had been rinsed three times with PBS. A Leica SP8 confocal fluorescence microscope was applied to view these samples (Leica Microsystems, Biberach, Germany).

### 2.9. Statistical Analysis

Statistical analysis was performed using Prism 7.0 software (GraphPad Software, San Diego, CA, USA). All quantitative experiments were repeated independently at least three times. Statistical differences between the three groups were examined with one-way analysis of variance (ANOVA), followed by Bonferroni corrections for multiple comparisons. *p* values < 0.05 were considered statistically significant.

## 3. Results

### 3.1. Subjects and Variant Identification

**Figure 1:**
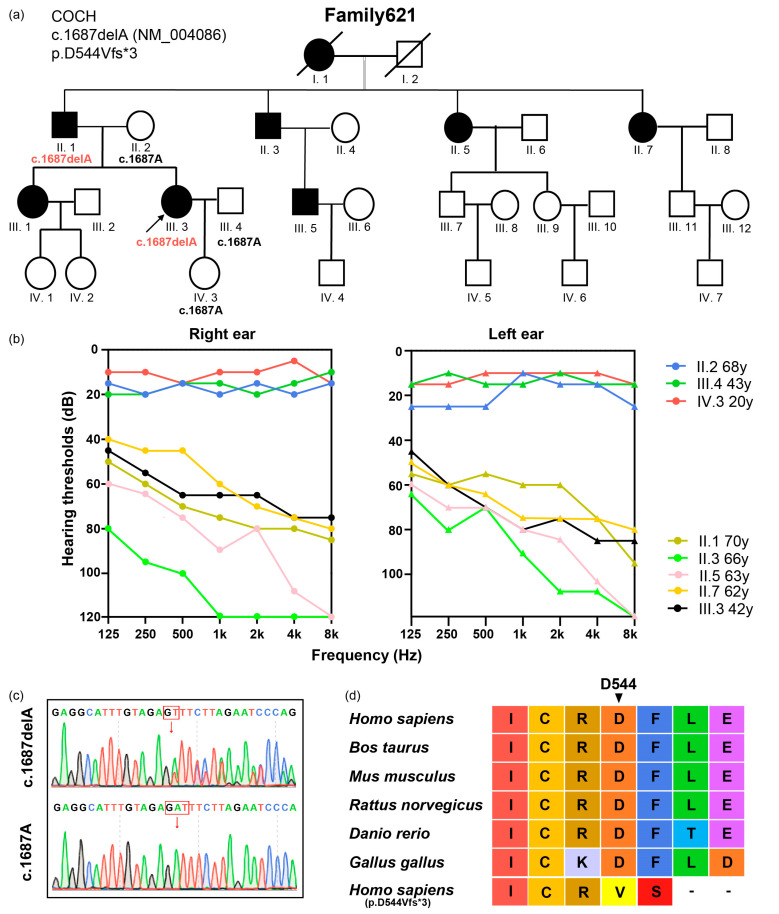
Identification of a *COCH* variant in Family621 with hearing loss. (**a**,**b**) Pedigree and air conduction audiograms; (**c**) representative Sanger sequencing chromatograms showing the c.1687delA variant; (**d**) conservation analysis of the variant site (p.D544) in *COCH* protein.

Family621 is a consanguineous Chinese family with four generations (Figure 1a). Hearing loss in Family621 is down-sloping moderately severe to severe bilateral sensorineural hearing loss (Figure 1b). All the affected family members were in their early 30 s at the onset of hearing loss. The proband, III.3, reported occasional dizziness, but no vertigo or balance problems. The dizziness occurred when the proband changed posture from sitting or lying to standing and the occurrence of dizziness was not related to the progression of hearing loss. Considering the existence of her vestibular symptom, III.3 was ordered with a whole set of vestibular function assessments. The neurotologic examinations showed normal videonystagmography. The caloric testing revealed a 22% UW on the left side, while the DP was below 25%. The otolithic organs of the utricle and saccule were evaluated by adapting the c-VEMP neuroelectrophysiological testing approach. The proband did not show any recognizable VEMP wave at 95 dB on the left side. On the right side, VEMP could be elicited with stimulations above the 75 dB threshold, P1, and the N1 latency was 17.61 ms and 25.94 ms, respectively, which was within the normal range in our hospital. Apart from those two abnormal results suggesting left-side vestibular dysfunction, the rest of the vestibular function assessments, i.e., the rotary chair test, vHIT, and SOT, revealed no substantial abnormalities. The gender (female) and age (44 years at the assessment time) of the proband were taken into consideration, and the exceptional abnormalities should be interpreted prudently, especially the c-VEMP result [36,37].

Next-generation sequencing was used to identify possible genetic causes of hearing loss in Family621, and Sanger sequencing was adopted to validate those variants. A novel *COCH* variant c.1687delA:p.D544Vfs*3 (NM_004086) in exon 12 was identified, which co-segregated with the hearing loss phenotype (Figure 1c). The identified variant is not found in ExAC, 1000G, or dbSNP databases, occurs in a highly conserved residue (Figure 1d), and has been predicted to be deleterious by the computational tool Mutation Taster [38] (Table 1).

### 3.2. 3D Protein Modeling

**Figure 2:**
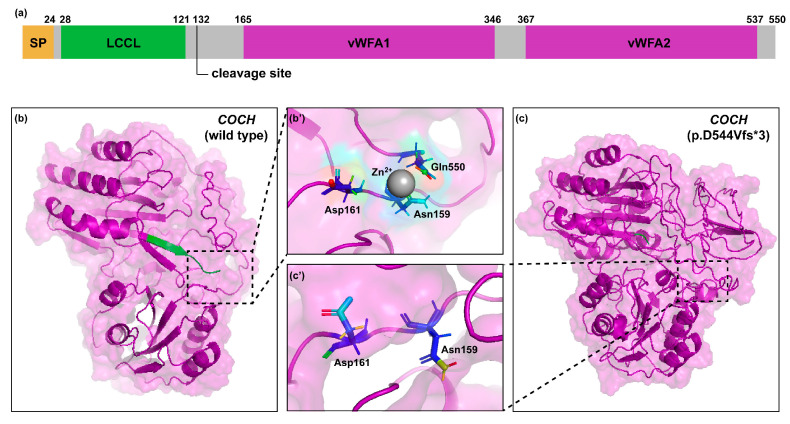
Molecular model of *COCH* protein. (**a**) Domain structure of *COCH* protein; (**b**,**c**) predicted 3D structures of *COCH* wild type and p.D544Vfs*3 mutant protein.

The p.D544 residue was located in a predicted helix, which was destroyed after the p.D544Vfs*3 variant (Figure 2b,c). Molecular visualization of the C-terminus of *COCH* showed that Gln550 was a predicted metal ion-binding site. The other two amino acid residues which participated in binding of the zinc ion, Asn159 and Asp161, were located in the intervening domain near the cleavage site of *COCH* protein. The frameshift variant and resulted premature termination may affect the interaction with zinc ion and the cleavage of mutant cochlin.

### 3.3. Molecular Basis of Genotype-Phenotype Correlation in COCH

**Figure 3:**
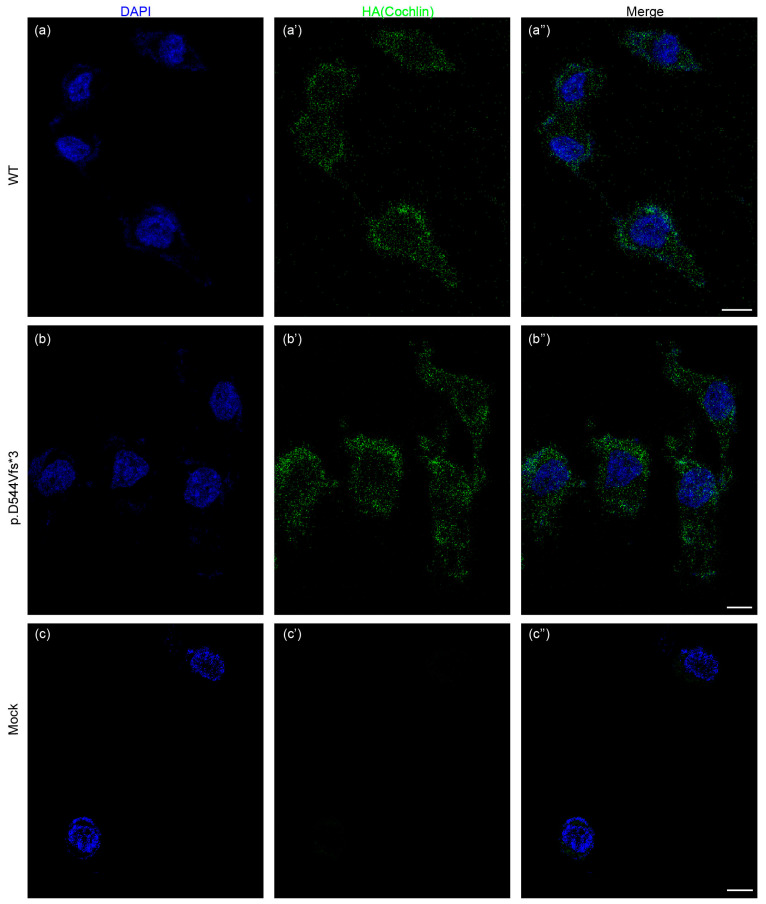
(**a**–**a”**) Immunofluorescence staining of the HEK293 cells transiently transfected with mutant *COCH* plasmid, (**b**–**b”**) wild-type *COCH* plasmid, (**c**–**c”**) or liposome vehicle using anti-HA antibody and stained with Alexa Fluor 488. Scale bar = 10 µm.

To investigate the effect of *COCH* variant on HEK 293T cell line, we transfected HEK 293T cells with wild-type or mutant *COCH* plasmid (Figure S1). Intracellular localization of wild-type and mutant cochlin proteins was compared in HEK 293T cells using immunofluorescence, in which cells were immunostained with anti-HA antibody (Figure 3a–c). Wild-type cochlin was located surrounding the nuclei, which were stained with DAPI, and its distribution was quite even. However, there was no obvious difference in the pattern of p.D544Vfs*3 variant cochlin location.

**Figure 4:**
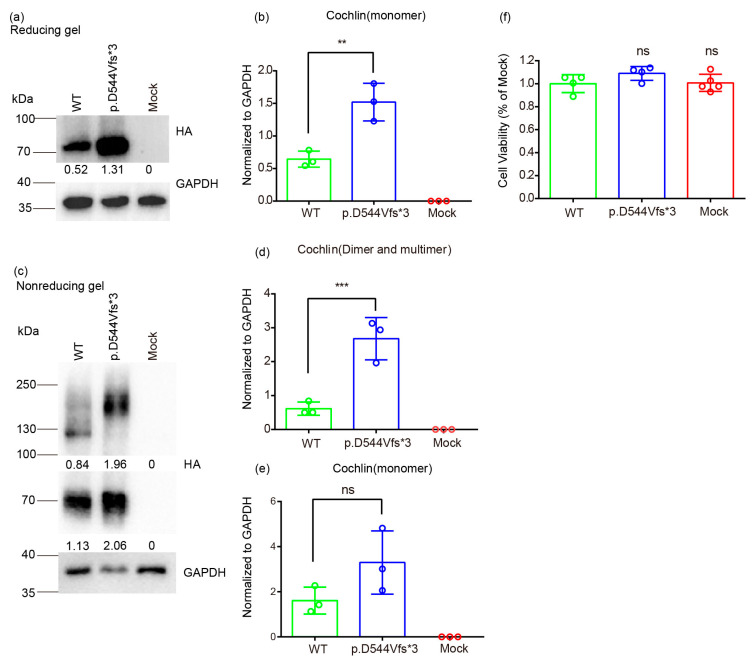
(**a**,**b**) Western blot results under reducing condition. (**c**–**e**) Western blot results under non-reducing condition. Intensity of the HA (cochlin) bands was quantified and normalized to the intensity of the GAPDH bands. (**f**) CCK8 assay result. Data represent mean ± standard deviation. **, *p* < 0.01; ***, *p* < 0.005; ns—not significant according to one-way ANOVA with Bonferroni correction for multiple comparisons to the reference of WT.

Western blot under reducing and non-reducing conditions was also performed to evaluate transfection efficiency and to compare intracellular cochlin levels, with HA being targeted as an indicator (Figure S2). As shown in Figure 4a, wild-type and mutant cochlin were both successfully expressed in cells. Notably, mutant cochlin in cell lysate was significantly higher than the wild-type cochlin, detected as monomer bands around 70 kDa with stronger intensity (Figure 4b). Under the non-reducing condition, likewise, a stronger band of cochlin monomer was observed in the mutant group (Figure 4c). Dimeric cochlin band was presented around 130kDa in the wild-type group while the variant group showed increased multimeric cochlin bands of a larger size (>130 kDa) (Figure 4d,e), which indicated a higher tendency to form aggregates.

Then, we tested the effect of *COCH* variant on cell viability in HEK 293T cells using CCK-8 assay. As shown in Figure 4f, cell viability was not significantly lower in mutant-*COCH*-transfected cells compared with those in wild-type cells. These results suggested that despite showing signs of intracellular cochlin accumulation, the p.D544Vfs*3 variant does not affect the viability of HEK 293T cells.

## 4. Discussion

In this study, we identified a novel frameshift variant at *COCH* in a big Chinese family with late-onset ARNSHL. The variant co-segregated with the disease and has not been observed in public databases before.

The p.D544Vfs*3 variant identified in this study resides near the C-terminal of cochlin, which resulted in several amino acid substitutions and premature translational termination. It has been shown that a nearby amino acid, 542 Cys, was critical to the disulfide bond formation and structural integrity of cochlin [22]. Three distinct variants of C542 have been reported to cause DFNA9 disorder [22,23,41]. It is possible that, in our case, the amino acid change at position 544 from aspartic acid (an acidic amino acid) to valine (a branched-chain amino acid) may also interfere with the protein folding and disrupt the normal structure of cochlin.

DFNA9 hearing loss occurs with different pathogenic mechanisms depending on variant location. Several studies suggested that amino acid substitutions near the cleavage site of cochlin caused dysfunctions in the cleavage of cochlin and the formation of extracellular matrix [39,40]. In our study, the binding ligand prediction results showed that Gln550, Asn159, and Asp161 were located close in the tertiary structure and formed a zinc ion binding site. The cleavage site of *COCH* was recognized by aggrecanase, a kind of zinc-dependent metalloprotease [43]. Variants located near the cleavage site and zinc-binding residues share similar phenotypes and biomolecular basis because they both disrupt the cleavage of cochlin by aggrecanase. However, zinc-binding residues, especially Asn159 and Asp161, are adjacent to C162, a conserved residue of cochlin, which can form disulfide bonds. This nature endows variants located near zinc-binding residues, such as p.C162Y, with serious misfolding, intracellular aggregation, and impaired protein trafficking compared to those of variants located near the cleavage site, like p.V123E [39]. Given that the p.D544Vfs*3 variant resulted in a lack of Gln550 due to earlier translation termination and potentially interfered with C542, it is possible that the p.D544Vfs*3 variant in this study also affects the post-translational cleavage of the cochlin and causes protein misfolding, which provides a potential explanation for the dominant nature of DFNA9 hearing loss.

Consistent with previous research concerning variants near the C-terminus of the vWFA2 domain, the p.D544Vfs*3 variant also resulted in more multimeric formations of cochlin and was regarded as more likely to cause relatively early-onset hearing loss, in reference to the DFNA9 spectrum, rather than severe vestibular symptoms [44]. The clinical manifestations of our proband, III.3, which showed mild vestibular symptoms accompanied by negative results in nearly all vestibular function assessments, were compatible with our molecular basis findings. Generally speaking, most of the affected subjects in Family621 did not manifest vestibular symptoms; this phenomenon was quite similar to that of subjects affected by the other C-terminal *COCH* variants (p.I541F, p.C542R, p.C542Y, and p.C542F [21,22,23,41]). However, the onset of hearing loss in p.D544Vfs*3 variant carriers was relatively late compared to those of the other C-terminal *COCH* variants (which varied from childhood to 20s), which may be due to its relatively greater distance from the critical C542 residue.

Some DFNA9 patients with *COCH* mutations located in the LCCL domain share manifestations assembling Meniere’s disease [45]. However, clear clinical differences existed between our subjects and a familial Meniere’s disease scenario. First, only III.3 had vestibular symptoms which had more correlation with posture changes rather than the progression of hearing loss. Second, DFNA9 patients show bilateral progressive deterioration of sensorineural hearing loss starting from high frequencies and then gradually progressing to all frequencies, unlike Meniere’s disease patients, who manifest fluctuating sensorineural hearing loss starting from low frequencies. This difference in hearing loss progression is likely caused by the different pathogenies of these two diseases. In this condition, Family621 should be distinguished from a familial Meniere’s disease. On the other hand, it is interesting that no matter how mildly vestibular symptoms have manifested in the subjects, c-VEMP abnormalities may exist. Every p.C542F, p.C542Y, and p.C162Y variant carrier who had taken the tests showed abnormal VEMP results, even though most of them had not complained of vertigo or dizziness. A study of vestibular phenotype based on a large series of p.P51S carriers has shown that c-VEMP is the quickest to deteriorate compared with vHIT and caloric responses [46]. Thus, the c-VEMP test may be a sensitive indicator for DFNA9. Further investigations are needed to verify whether it could help with DFNA9 diagnosis among those with atypical symptoms.

The cell viability assay demonstrated no cytotoxicity of the p.D544Vfs*3 variant in HEK 293T cell line. According to previous research, not every mutant cochlin possesses a cytotoxic nature [5]. To date, only p.Gly90Glu and p.Phe230Leu mutant cochlin have shown cytotoxicity towards UB/UE-1 cells, primary fibrocytes derived from the cochlear and NIH 3T3 cell line, respectively [5,19]. It is reasonable to speculate that cytotoxicity is not the actual way the p.D544Vfs*3 variant causes hearing loss.

Our research had several limitations. Although the p.D544Vfs*3 variant was predicted to affect the cleavage of cochlin and the release of the LCCL domain, we did not verify this hypothesis in the present study by conducting Western blot with a medium protein sample to observe the amount of cleaved and noncleaved cochlin [18,39]. Only the ELISA was taken to measure cochlin in culture media from different groups, and the results showed that the variant did not affect the whole amount of cochlin secretion. LCCL plays a crucial part in innate immunity during bacteria-induced inflammation in the cochlea [47]. It has been reported that a deficit in secretion of the LCCL peptide promotes pathogen-induced cochlear damage while decreasing inflammatory cytokine expression and phagocyte recruitment [18,48]. Given these findings, the LCCL peptide should be regarded as a potential target in our further research. Measurement of LCCL peptide, cleaved and noncleaved cochlin collected from culture medium should be taken into consideration in order to testify its impaired secretion due to the p.D544Vfs*3 variant. Apart from the impaired secretion from the cell, cochlin multimerization may be related to failure of protein trafficking from ER to Golgi [44]; thus, ER and Golgi immunostaining should be performed to analyze the colocalization of p.D544Vfs*3 variant cochlin. Furthermore, mutant cochlin secreted by HEK 293T cells is actually unlikely to show cytotoxicity towards those cells themselves, even though its cytotoxic nature can be proven elsewhere by intracochlear injection or incubating primary fibrocytes with conditioned medium from 293T cells [19]. In the present study, we did not use any inner ear-originating cells for protein multimerization or for the cytotoxicity assay. To clarify whether the p.D544Vfs*3 variant impairs hearing through a cytotoxic mechanism, we propose testing it with a conditioned medium intracochlear injection and in vitro experiments like primary inner ear cell culturing in the future. An animal model of *COCH* p.D544Vfs*3 variant knock-in mice may be the most valid way to fully demonstrate possible mechanisms. Histological morphology variations and immune phenomena can be observed during the onset and deterioration of hearing in the animal model.

Last but not least, audiometric monitoring of all family members should be conducted, especially for those young family members in Generation VI who are presently around their 20s. This can, on one hand, help clinical practitioners to intervene as soon as possible and, on the other hand, provide more information about hearing loss progression of DFNA9 caused by the novel *COCH* p.D544Vfs*3 variant. It has been reported that patients treated with cochlear implants for DFNA9 hearing loss show considerable improvements in postoperative word recognition scores which were comparable to other cochlear implant patients with other progressive hearing losses [49]. Cochlear implants may act as a potential therapeutic option for DFNA9 patients, at least regarding intervention for hearing loss. It could be especially beneficial to p.D544Vfs*3 and the other C-terminal *COCH* variant carriers because it seems that non-syndromic hearing loss is the only stably manifested detriment.

## 5. Conclusions

In conclusion, our findings enriched the phenotypic and variant spectrum of DFNA9 in Chinese people and implied the importance of the diagnosis of the *COCH* gene in patients with late-onset hereditary hearing loss. A *COCH* variant located near the C-terminus of the vWFA2 domain can lead to multimeric cochlin formation and relatively early-onset hearing loss, while vestibular symptoms may not be present.

## Figures and Tables

**Table 1:** Novel and previously reported DFNA9 variants in the non-domain region of *COCH* protein.

Pedigree	Ethnicity	Age of Onset	Mode	g.DNA	c.DNA ^a^	Protein	MutationTaster Prediction (Prob) ^b^	HL-Specific ACMG Classification	HL-Specific ACMG Components	CADD ^c^	Novelty (Reported PMID)
YUHL5	Korean	37–52 years	AD	14:31348145T>A	c.424T>A	p.V123E	Disease causing (1.00)	Likely pathogenic	PS3, PM2, PP1, PP3, PP4	26.8	26256111 [39]
Family32, Family208	Chinese	12–67 years	AD	14:31349796G>A	c.541G>A	p.C162Y	Disease causing (1.00)	Likely pathogenic	PS3, PM2, PP1, PP3, PP4	28.1	28099493 [40]
-	Caucasian	childhood	-	14:31358965A>T	c.1677A>T	p.I541F	Disease causing (1.00)	Likely pathogenic	PS3, PM2, PP4	26.3	31493294 [21]
FAM986	Japanese	childhood	AD	14:31358968T>C	c. 1680T>C	p.C542R	Disease causing (1.00)	Likely pathogenic	PM2, PM5, PP1, PP3, PP4	24.6	25780252 [41]
HL3	European	childhood	AD	14:31358969G>T	c.1681G>T	p.C542F	Disease causing (1.00)	Likely pathogenic	PS3, PM2, PP1, PP3, PP4	24.1	16261627 [22]
SD-Z001	Chinese	15–59 years	AD	14:31358969G>A	c.1681G>A	p.C542Y	Disease causing (1.00)	Likely pathogenic	PM2, PM5, PP1, PP3, PP4	24.0	18312449 [23] 18269866 [42]
Family621	Chinese	early 30s	AD	14:31358975_ 31358975delA	c.1687delA	p.D544Vfs*3	Disease causing (1.00)	Likely pathogenic	PM2, PM4, PP1, PP4	-	This study

Abbreviations: AD—autosomal dominant; HL—hearing loss; ACMG—American College of Medical Genetics and Genomics. ^a^ Variants are numbered according to NCBI human cDNA reference sequence NM_004086.2. ^b^ MutationTaster (https://www.mutationtaster.org/, accessed on 5 December 2021) predicts a variant as one of four possible types: disease causing automatic, disease causing, polymorphism, and polymorphism automatic. ^c^ PHRED-like scaled C-scores on Combined Annotation-Dependent Depletion (https://cadd.gs.washington.edu/snv), a score ≥ 20 indicates the 1% most deleterious substitutions to the human genome.

## Data Availability

The data underlying this article will be shared on reasonable request to the corresponding author.

## Supplementary Materials

The following supporting information can be downloaded at: https://www.mdpi.com/article/10.3390/life14010033/s1, Figure S1: Design of wild-type and mutant *COCH* plasmids. Figure S2: Uncropped whole blots. Table S1: The *COCH* variants associated with DFNA9.
