# Supplementary material for: A Novel COCH p.D544Vfs*3 Variant Associated with DFNA9 Sensorineural Hearing Loss Causes Pathological Multimeric Cochlin Formation
Source: Life (Basel). 2023 Dec 25;14(1):33. doi: 10.3390/life14010033 (PMC10817332; doi:10.3390/life14010033)
Supplement: Supplementary file 1 [file life-14-00033-s001.zip › life-2734801-supplementary.pdf]

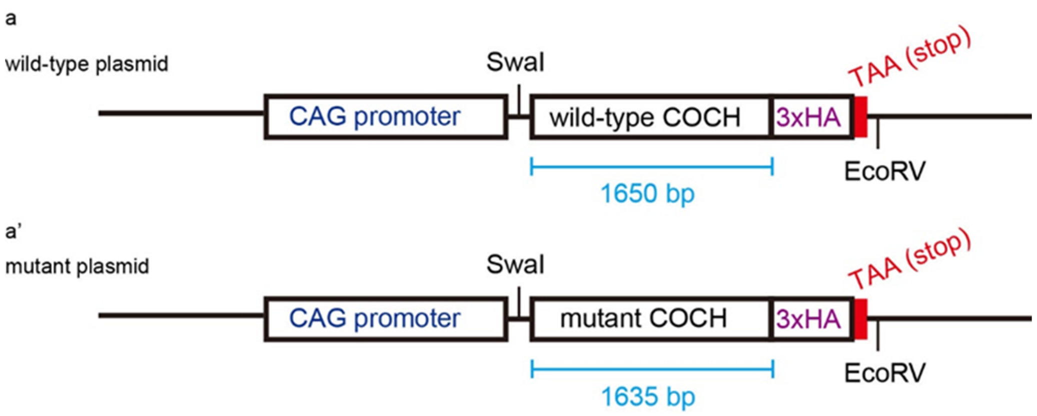

Figure S1. (a) Design of wild-type and (a') mutant COCH plasmids, the 3xHA fragment was inserted before the COCH stop codon TAA (red).

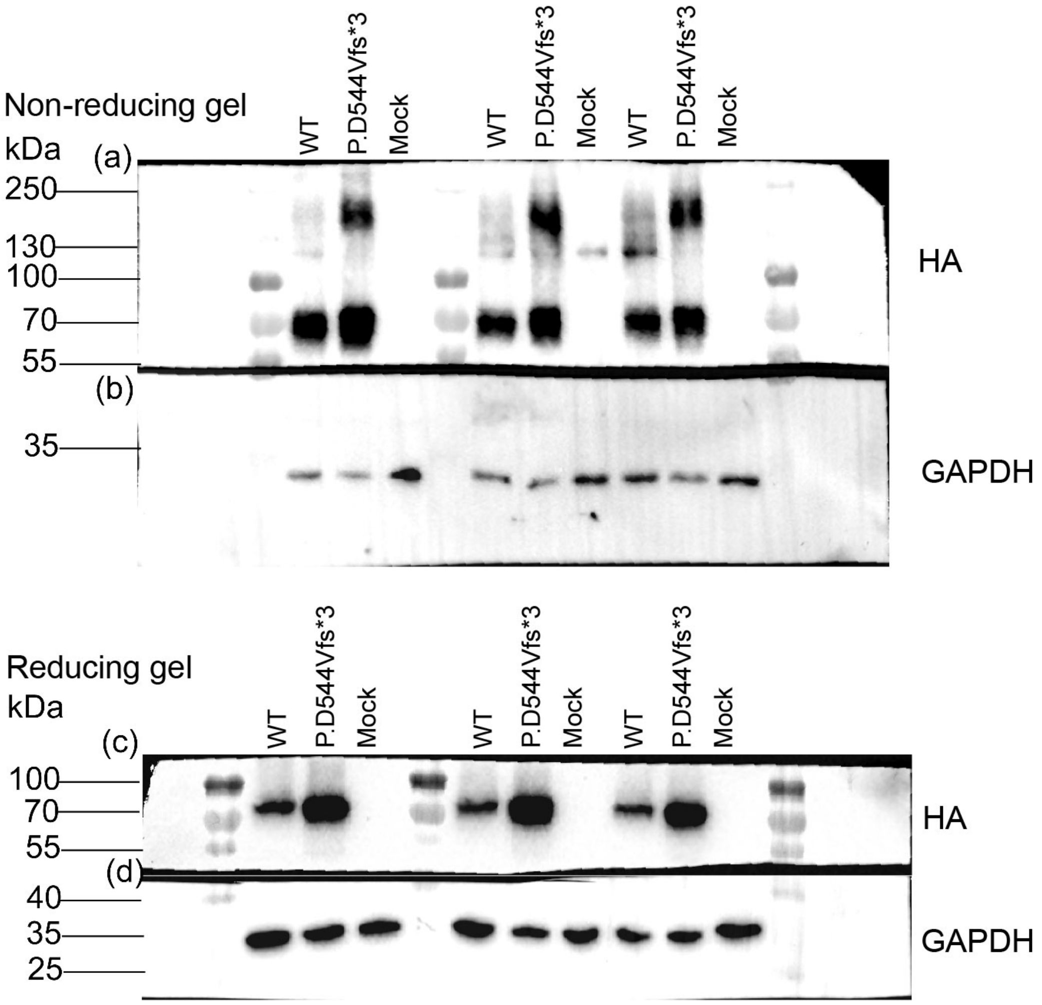

Figure S2. (a-b) Uncropped result of non-reducing western blot; (c-d) Uncropped result of reducing western blot. The same samples were run in triplicate under both conditions.

Table S1. The COCH variants associated with DFNA9

Pedigree/<br>Author year	Ethnicity	Age of onset	Mode	g.DNA (GRCh37)	c.DNA [a]	Protein	MutationTaster prediction (prob) [b]	CADD[c]	Novelty (reported PMID/doi)
JSNY-027<br>SH14<br>SH140<br>SB200	Chinese<br>Korean	30s/40s/late onset	AD	14: 31346808 G>A	c.113G>A	p.G38D	Disease-causing	25.8	25388789<br>26758463<br>25279224
Hildebrand et al, 2009	American	30s-70s	AD	14: 31346846 C>T	c.151C>T	p.P51S	Disease-causing	26.4	19161137
Family AD1	Austrian								28733840
Fransen et al, 1999	Belgian								10400989
Verstreken et al, 2001									11698812
McComiskey et al, 2010	Canadian								-
de Varebeke, S.P.J. et al, 2014	Caucasian								24662630
Verhagen et al, 2001<br>Bischoff et al, 2005	Dutch								2788715<br>11843927
kindred 1W	American	10s-20s	AD/NA	14: 31346892 T>G	c.197T>G	p. V66G	Disease-causing	25.4	10942145<br>9806553
Sloan-Heggen et al, 2016	Caucasian	Teens - 40	AD	14:31346921 G>A	c.226G>A	p.A76T	Disease-causing	27.0	26969326

Family W05-196	Dutch	10s-60s	AD	14:31348036 G>T	c.259G>T	p.G87W	Disease-causing	32	16835921 17264471
Family D882	Chinese	Mid–40s	AD	14:31348037 G>T	c.260G>T	p.G87V	Disease-causing	27.3	23993205
kindred 1Su<br>Family W99-101<br>Family 368	American<br>Dutch<br>Japanese	40s-60s	AD	14:31348040 G>A	c.263G>A	p.G88E	Disease-causing	27.7	9806553<br>16151339<br>25780252
Family G405	Chinese	20s-30s	AD	14:31348052T >A	c.275T>A	p.V92D	Disease-causing	24.5	28116169
Nagy et al, 2005	Hungarian	30s	NA	14:31348088_31348090delTAG	c.311_313delTAG	p.V104del	polymorphism	-	14729849
Pauw et al, 2011<br>Kamarinos et al, 2001	Australian	20s-40s	NA	14:31348103T >A	c.326T>A	p.I109N	Disease-causing	28.0	21774451<br>11295836
Family W05-427	Dutch	30s-50s	AD	14:31348103T >C	c.326T>C	p.I109T	Disease-causing	24.3	17561763
Burgess et al, 2016<br>SB50	American<br>Korean	Late 20s-30s	AD	14:31348118T >C	c.341T>C	p.L114P	Disease-causing	29.2	27023102<br>23990876

kindred 1St<br>Baek et al, 2010	American<br>Korean	Early 30s	AD	14:31348126T<br>>C	c.349T>C	p. W117R	Disease-<br>causing	29.3	9806553<br>20447147
Usami et al, 2003	Japanese	40s	AD	14:31348132<br>G>A	c.355G>A	p.A119T	Disease-<br>causing	22.4	14512963
Family 467	American	20s-30s	AD	14:31348139T<br>>C	c.362T>C	p.F121S	Disease-<br>causing	31.0	21046548
YUHL5	Korean	Late 30s-<br>50s	AD	14:31348145T<br>>A	c.368T>A	p.V123E	Disease-<br>causing	26.8	26256111
Family #208<br>Family #32<br>SB82	Chinese<br><br>Korean	20s-40s	AD	14:31349796<br>G>A	c.485G>A	p.C162Y	Disease-<br>causing	28.1	28099493<br>22931125<br>25830873<br>26758463
Oziebło et al, 2018<br>Family 475	Polish<br>Japanese	10s-40s	AD	14:31355156T<br>>C	c.1115T>C	p.I372T	Disease-<br>causing	26.9	doi:10.17430/1002<br>738.<br>25780252
Family W08-2035	Dutch	Late 10s-<br>40s	AD	14:31355353C<br>>T	c.1312C>T	p.R438C	Disease-<br>causing	23.9	33710989
Gallant et al, 2013	American	Mid to late<br>20s	AD	14:31355237_<br>31355254delT<br>CTCGGACA<br>TTGGTGCC<br>A	c.1196_1213d<br>el18	p.I399_A404<br>del	Disease-<br>causing -<br>long InDel	-	23374487

Faletra et al, 2011	Italian	20s-30s	AD	14:31355500 G>C	c.1459G>C	p.A487P	polymorphism	24.0	doi:10.3109/1651386X.2010.538523
HLJ-Z079	Chinese	40s	AD	14:31358879T>C	c.1535T>C	p.M512T	Disease-causing	23.9	18312449
SD-39	Korean	Childhood	AD	14:31358924T>G	c.1580T>G	p.F527C	Disease-causing	29.5	22610276
Basu et al, 2019	Caucasian	Childhood	AD	14:31358965 A>T	c.1621A>T	p.I541F	Disease-causing	26.3	31493294
Family 986	Japanese	Grade school	AD	14:31358968T>C	c.1624T>C	p.C542R	Disease-causing	24.6	25780252
Family SD-Z001	Chinese	20s-50s	AD	14:31358969 G>A	c.1625G>A	p.C542Y	Disease-causing	24.0	18312449
HL3 Sloan-Heggen et al, 2016	American Caucasian	10s-20s	AD	14:31358969 G>T	c.1625G>T	p.C542F	Disease-causing	24.1	16261627 26969326

Abbreviations: AD, autosomal dominant; HL, hearing loss; ACMG, American College of Medical Genetics and Genomics

a. Variants are numbered according to NCBI human cDNA reference sequence NM_004086.2

b. MutationTaster (https://www.mutationtaster.org/) predicts a variant as one of four possible types: disease-causing automatic, disease-causing, polymorphism, and polymorphism automatic

c. PHRED-like scaled C-scores on Combined Annotation-Dependent Depletion (https://cadd.gs.washington.edu/snv), a score ≥ 20 indicates the 1% most deleterious substitutions to the human genome
